# EGCG Attenuates Autoimmune Arthritis by Inhibition of STAT3 and HIF-1α with Th17/Treg Control

**DOI:** 10.1371/journal.pone.0086062

**Published:** 2014-02-18

**Authors:** Eun-Ji Yang, Jennifer Lee, Seon-Young Lee, Eun-Kyung Kim, Young-Mee Moon, Young Ok Jung, Sung-Hwan Park, Mi-La Cho

**Affiliations:** 1 The Rheumatism Research Center, Catholic Research Institute of Medical Science, The Catholic University of Korea, Seoul, South Korea; 2 Center for Rheumatic Disease, Division of Rheumatology, Department of Internal Medicine, Seoul St. Mary's Hospital, The Catholic University of Korea, Banpo-dong, Seocho-gu, Seoul, South Korea; 3 Laboratory of Immune Network, Conversant Research Consortium in Immunologic Disease, Seoul St. Mary's Hospital, College of Medicine, The Catholic University of Korea, Seoul, South Korea; 4 Division of Rheumatology, Department of Internal Medicine, Hallym University Kang-Nam Sacred Heart Hospital, Seoul, Korea; Wayne State University, United States of America

## Abstract

Epigallocatechin-3-gallate (EGCG) is a green tea polyphenol exerting potent anti-oxidant and anti-inflammatory effects by inhibiting signaling and gene expression. The objective of the study was to evaluate the effect of EGCG on interleukin (IL)-1 receptor antagonist knockout (IL-1RaKO) autoimmune arthritis models. IL-1RaKO arthritis models were injected intraperitoneally with EGCG three times per week after the first immunization. EGCG decreased the arthritis index and showed protective effects against joint destruction in the IL-1RaKO arthritis models. The expression of pro-inflammatory cytokines, oxidative stress proteins, and *p*-STAT3 (Y705) and *p*-STAT3 (S727), mTOR and HIF-1α were significantly lower in mice treated with EGCG. EGCG reduced osteoclast markers *in vivo* and *in vitro* along with anti-osteoclastic activity was observed in EGCG-treated IL-1RaKO mice. The proportion of Foxp3^+^ Treg cells increased in the spleens of mice treated with EGCG, whereas the proportion of Th17 cells reduced. *In vitro*, *p*-STAT3 (Y705) and *p*-STAT3 (S727), HIF1α and glycolytic pathway molecules were decreased by EGCG. EGCG suppressed the activation of mTOR and subsequently HIF-1α, which is considered as a metabolic check point of Th17/Treg differentiation supporting the therapeutic potential of EGCG in autoimmune arthritis.

## Introduction

Rheumatoid arthritis (RA) is a systemic autoimmune disease characterized by chronic inflammation of multiple joints that can result in joint destruction if left untreated. Although the exact pathogenic mechanism remains elusive, T cells abundant in the synovial milieu are in the center of pathogenesis orchestrating innate and adaptive immune responses. The cytokine milieu that is enriched with interleukin (IL)-1β, IL-6, and IL-23 provides Th17 polarizing condition in the synovium of arthritic joint. IL-17 that is mainly produced by Th17, promotes the activation of synoviocytes and chondrocytes in concert with tumor necrosis factor (TNF)- α, enhancing osteoclast formation, which is essential in bone erosion [1]. On the other hand, the function of Treg is known to be suppressed by TNF-α in RA [2]. Therefore, numerous efforts have been made to treat RA by targeting Th17 while enhancing Treg. Recent advance in understanding of Th17 development revealed that mammalian target of rapamycin (mTOR) induced hypoxia-induced factor (HIF)-1α determined the fate of T cell whether it differentiated into Th17 or Treg [3], suggesting mTOR-HIF-1α as a promising target for RA treatment.

Green tea is one of the most popular beverages in the world, which is believed to exert beneficial effects on health and disease. The catechins including epicatechin, epigallocatechin, epicatechin-3-gallate, and epigallocatechin-3-gallate (EGCG) are the major components of green tea. Among them, EGCG is the most biologically active component that is responsible for the most of the pharmacologic effect of green tea. EGCG functions as a powerful antioxidant preventing oxidative damage to various types of healthy cells. This has also shown to suppress angiogenesis in tumor cells showing anti-cancer effect [4].

Animal studies revealed that EGCG ameliorated inflammatory arthritis in collagen-induced arthritis (CIA) model [5] and adjuvant-induced arthritis model [6]. The expression of proinflammatory cytokines in the serum and the joints were significantly reduced with oral or intraperitoneal administration of EGCG. In addition, EGCG has been reported to suppress osteoclastogenesis by inhibiting downstream signaling of receptor activator nuclear factor kappa B (NFκB) (RANK) implying its role in preventing bone erosion in RA. However, a direct link showing the regulation of EGCG on pathogenic T cell in autoimmune arthritis is still lacking. Acknowledging the essential role of T cells in RA, a study focused on the effect of EGCG on T cell subset in arthritic conditions seemed to suggest its therapeutic potential. In the present study, the anti-arthritic effect of EGCG in IL-1 receptor antagonist knockout (IL-1RaKO) CIA mice was demonstrated. Furthermore, this study illustrated EGCG regulation on Th17/Treg differentiation via the regulation of mTOR and HIF-1α.

## Materials and Methods

### Animals

IL-1RaKO mice in the BALB/c background were kindly provided by Y Iwakura (University of Tokyo) and were maintained under specific-pathogen-free conditions at the Institute of Medical Science, Catholic University of Korea. IL-1RaKO mice were fed standard mouse chow (Ralston Purina, St. Louis, MO) and water ad libitum. All experimental procedures were examined and approved by the Animal Research Ethics Committee of the Catholic University of Korea (permit number: CUMC-2010-0011-03), which conforms to all National Institutes of Health of the USA guidelines. All surgeries were performed under isoflurane anesthesia, and all efforts were made to minimize suffering.

### EGCG treatment

EGCG was kindly provided by Hanlim Pharmaceutical Company (Seoul, Korea). EGCG dissolved in saline. The mice were intraperitoneally injected with EGCG (40 mg/kg) three times per week for 2.5 weeks. The control mice were injected with saline. Blood samples were collected from all treated and control mice 8 weeks after the primary immunization and stored at −70°C until use.

### Clinical assessment of arthritis

The severity of arthritis was determined by three independent observers. The mice were observed three times a week for the onset and severity of joint inflammation. The severity of arthritis was assessed on a scale of 0–4 with the following criteria, as described previously [7]: 0 =  no edema or swelling, 1 =  slight edema and erythema limited to the foot or ankle, 2 =  slight edema and erythema from the ankle to the tarsal bone, 3 =  moderate edema and erythema from the ankle to the tarsal bone, and 4 =  edema and erythema from the ankle to the entire leg. The arthritic score for each mouse was expressed as the sum of the scores of three limbs. The highest possible arthritis score for a mouse was thus 12. The mean arthritis index was used to compare the data among the control and experimental groups.

### Histopathology of arthritis

The mouse joint tissues were fixed in 4% paraformaldehyde, decalcified in EDTA bone decalcifier, and embedded in paraffin. Seven-micrometer sections were prepared and stained with H&E, Safranin O, and toluidine blue to detect proteoglycans. The sections were dewaxed using xylene; then they were dehydrated in a graded series of alcohols. The endogenous peroxidase activity was quenched with methanol and 3% H_2_O_2_. Immunohistochemistry (IHC) was performed using the Vectastain ABC kit (Vector Laboratories, Burlingame, CA). The tissues were first incubated with the primary anti-VEGF, anti–TNF-a, anti–IL-1β, anti–IL-17, anti-RANK (all from Santa Cruz Biotechnology, Santa Cruz, CA), anti–IL-6 (Abcam), anti-mTOR, anti-STAT3 (Cell signaling), anti-HIF-1α (NOVUS), goat IgG isotype (for TNF) or rabbit IgG isotype (for IL-1β, IL-6, IL-17, RANK, VEGF, HIF-1α, mTOR and STAT3) overnight at 4°C; and a biotinylated secondary linking Ab and a streptavidin–peroxidase complex for 1 h. The final color product was developed using 3,3-diaminobenzidine chromogen (DAKO, Carpinteria, CA). The sections were counterstained with hematoxylin. Tartrate-resistant acid phosphatase (TRAP) staining was performed with a commercial kit (cat. no. 387-A, Sigma, St Louis, MO), according to the manufacturer's instructions, omitting counterstaining with hematoxylin. TRAP+ multinucleated cells with three or more nuclei were considered osteoclasts. The numbers of osteoclasts were determined according to the method described by Bendele *et al.* [8]. All histological assessments were determined by two independent blinded observers. Images were captured using a DP71 digital camera (Olympus, Center Valley, PA) attached to an Olympus BX41 microscope at 3400 magnification.

### CII-specific T cell response

The spleen was minced and the cells were filtered through a cell strainer and centrifuged at 1500 rpm at 4°C for 10 min. CD4+ T cells were purified by negative selection using the CD4^+^ T Cell Isolation Kit (Miltenyi Biotec, Bergisch Gladbach, Germany). The CD4^+^ T cells (2×10^5^/well) were cultured in 96-well plates at 37°C for 72 h in the presence of 0.5 µg/ml anti-CD3 mAb. The cells were cultured for 72 h, and 18 h before the termination of culture, 1 mCi [3H] thymidine (Amersham Pharmacia. Biotech, Little Chalfont, U.K.) were added to each well. The cells were harvested onto glass fiber filters, and the radioactivity incorporated in the cells was measured using a Wallac Betaplate liquid scintillation counter (Beckman, Fullerton, CA). The results were expressed as the mean cpm.

### Osteoclastogenesis

Bone marrow-derived monocyte/macrophage (BMM) cells were isolated from the tibia and femur of the mice. The cells were incubated with α-MEM (Invitrogen, Burlingame, CA) containing antibiotics and 10% heat inactivated FBS for 12 h to separate the floating cells and adherent cells. The floating cells were seeded on 48-well plates at 2×10^5^ cells/well, and they were cultured in the presence of 10 ng/ml recombinant human (rh) MCSF (R&D Systems, Minneapolis, MN) with α-MEM. Three days later, the washed out nonadherent cells and pre-osteoclasts were further cultured in the presence of 10 ng/ml M-CSF, 50 ng/ml RANK ligand (RANKL) (PeproTech, London, U.K.), and various concentrations of EGCG for 4 d, to generate osteoclasts. TRAP staining was performed. TRAP+ cells with three or more nuclei were defined as osteoclasts and the numbers of osteoclasts were counted.

### Confocal microscope

For immunostaining, 7 µm tissue sections of the spleens were stained using PE-conjugated anti-IL-1R, PE-conjugated anti-*p*-STAT3 (Y705), PE-conjugated anti-*p*-STAT3 (S727), PE-conjugated anti-IL-1R, PE-conjugated anti-IL-1β, FITC-conjugated anti-Foxp3, PE-conjugated anti-IL-17, APC-conjugated anti-CD25, FITC conjugated anti-CD4 (all from eBioscience, San Diego, CA) and with DAPI (Sigma) overnight at 4°C. The stained sections were analyzed using a Zeiss microscope (LSM 510 Meta; Carl Zeiss, Oberkochen, Germany) at 3400 magnification.

### Flow Cytometry

We investigated changes in the T-cell subpopulation after EGCG treatment. flow cytometry was performed using various combinations of fluorochrome-conjugated antibodies to CD4 (eBioscience), CD25 (BioLegend), Foxp3 (eBioscience), IL-1R (eBioscience), IL-17 (eBioscience), *p*-STAT3 705 (BD Bioscience), *p*-STAT3 727(BD Bioscience). Cytokine secretion was stimulated by PMA (25 ng/ml; Sigma-Aldrich) and ionomycin (250 ng/ml; Sigma-Aldrich) in the presence of Golgi-stop (1 µl/ml; BD Bioscience) in 5% CO_2_ at 37°C for 4 hours. A total of 1×10^6^ spleen cells were washed and re-suspended in FACS buffer (phosphate-buffered saline, 0.5% bovine serum albumin, 0.1% sodium azide). Total spleen cells were washed and stained with primary (surface) fluorochrome-conjugated antibodies. Cells were then incubated for another 30 min at 4°C with antibodies and washed twice with FACS buffer. Spleen cells were fixed and permeabilized using the BD Cytofix/Cytoperm Kit (BD Bioscience), and then stained with intracellular antibodies. The data were acquired using a FACS Calibur (BD Diagnostic System, Sparks, MD) and analyzed with the Flowjo software (TreeStar, San Carlos, CA)

### Measurement of immunoglobulin concentrations

The serum concentration of IgG2a and collagen-specific IgG2a were measured by ELISA using commercially available kits (Bethyl Laboratories, Montgomery, TX). To measure collagen-specific IgG2a levels, flat-bottomed plates were coated with bovine CII and incubated overnight at 4°C. Serially diluted serum samples were then loaded into the wells and incubated at room temperature for 1 h. The wells were then washed with washing buffer (PBS containing 50 mM Tris, 0.14 M NaCl and 0.05% Tween 20), followed by horseradish peroxidase (HRP)-conjugated goat anti-mouse IgG2a antibodies (Bethyl Laboratories). HRP activity was measured using tetramethyl benzidine (TMB; eBioscience, San Diego, CA). The OD in each well was measured at a 450 nm in an ELISA plate reader (Bio-Rad, Hercules, CA).

### Analysis of the gene expression by real-time quantitative PCR

Total RNA was extracted using TRIzol (Molecular Research Center, Cincinnati, OH). RNA concentrations were measured in a NanoDrop ND-1000 (Thermo Fisher Scientific, MA, USA). Two micrograms of total RNA was reverse-transcribed using the Transcriptor First Strand cDNA Synthesis Kit (Roche Applied Science). mRNA expression was estimated by real-time PCR with FastStart SYBR Green Master (Roche Applied Science) using StepOnePlus (Applied Biosystems) according to the manufacturer's instructions.

### Cell preparation and culture

The mouse spleens were collected for cell preparation and washed twice with PBS. The spleens were minced and the red blood cells were lysed with 0.83% ammonium chloride. The cells were filtered through a cell strainer and centrifuged at 1300 rpm at 4°C for 5 min. The cell pellets were resuspended in RPMI 1640 medium and plated in 24-well plates (Corning, NY, USA) at a concentration of 1×10^6^ cells/well. Splenic CD4^+^ T cells were and stimulated with 0.5 µg/ml plate-bound anti-CD3 mAb and 1 µg/ml anti-CD28 mAb (BD Pharmingen) for 3 days under Th17-polarizing condition (2 µg/ml anti-IFN-γ, 2 µg/ml anti-IL-4, 2 ng/ml transforming growth factor (TGF)- β, 20 ng/ml IL-6). After 3 days of stimulation, the cells were restimulated with 25 ng/ml PMA and 250 ng/ml ionomycin (both from Sigma, St. Louis, MO) in the presence of GolgiStop (BD Pharmingen) for 5 h.

### Measurement of TNF-α and IL-17 in culture supernatants

Isolated splenocytes were cultured for 72 h. The amounts of TNF-α, IL-17 and IL-10 in the culture supernatants were measured by ELISA. Antibodies directed against mouse TNF-α and IL-17 and against biotinylated anti-mouse TNF-α and IL-17 were used as the capture and detection antibodies, respectively. Alkaline phosphatase (Sigma) was used for the chromogenic reaction. The amounts of cytokines present in the test samples were determined from standard curves constructed with serial dilutions of recombinant murine TNF-α and IL-17 (R&D Systems). The absorbance was determined with an ELISA microplate reader at 405 nm.

### Western blotting

Freshly isolated splenocytes or IL-6 (20 ng/ml)-stimulated splenocytes from the mice treated with EGCG or untreated were washed with cold PBS, and the total proteins were extracted with lysis buffer (1% Nonidet P-40, PMSF, 2 mM sodium vanadate, 0.1% sodium deoxycholate, and protease inhibitor mixture; Roche Applied Science, Mannheim, Germany). The harvested lysates were centrifuged for 15 min at 4°C to pellet cellular debris. The supernatants were removed and stored at −70°C. The protein lysate (15 µg) was loaded on 10% SDS-PAGE, followed by transfer to nitrocellulose membranes (Invitrogen Life Technologies). The blots were then blocked with 5% nonfat dry milk in TBST for 1 h at room temperature. Then the blots were incubated overnight at 4°C with Abs specific for *p*-STAT3 (Y705), HIF-1α or mTOR, after washing with TBST, the blots were incubated with goat anti-mouse or anti-rabbit HRP-conjugated secondary Abs and the bands were revealed with ECL reagents (Amersham Biosciences, Piscataway, NJ). After stripping, the total anti-STAT3, anti-β-actin was used as a loading control.

### Statistical analysis

Results were calculated using the GraphPad Prism 4.0 software and are presented as the means ± SD of at least three experiments. A *P* value of <0.05 was considered to indicate statistical significance. Data were compared by two-factor ANOVA with Bonferroni's post-test or by the Mann-Whitney U-test, as appropriate.

## Results

### EGCG suppresses collagen-induced arthritis in IL-1RaKO mice

**Figure 1:**
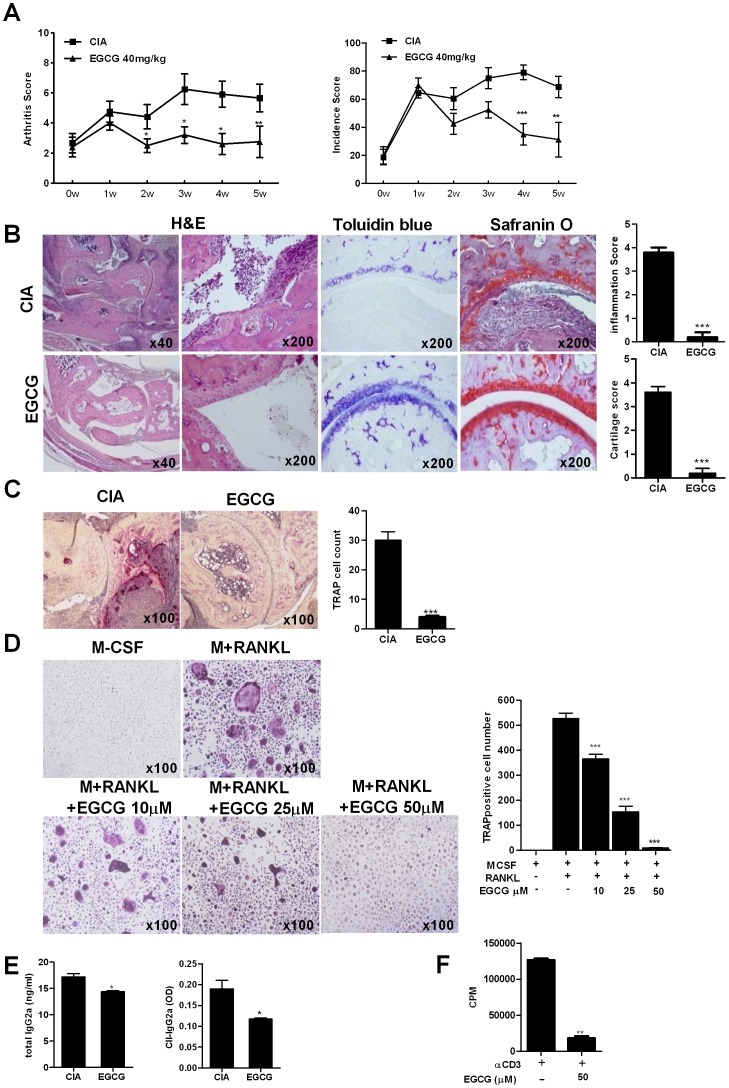
Treatment with EGCG suppresses inflammatory arthritis in IL-1RaKO mice. IL-1RaKO mice were immunized with 100 µg of Cll in CFA to induce arthritis. The mice were intraperitoneally injected with saline or EGCG (40 mg//kg) three times per week for 2.5 weeks. (A) Disease severity was recorded using the mean arthritis score ± SD (left) and arthritis incidence (right). (B) Tissue sections from the joints of each mouse were stained with H&E, toluidine blue and safranin O. (C) Tissue sections from the joints from the IL-1RaKO mice treated with EGCG (n = 10) or untreated (n = 10) were stained with TRAP. The histopathologic score of osteoclast formation is shown in the right graph. (D) TRAP staining for identification of osteoclasts. Osteoclast precursors were cultured in the presence of EGCG with M-CSF and RANKL. TRAP+ cells containing three or more nuclei were scored as osteoclast. TRAP+ cells were counted three times by blind scoring. (E) The levels of IgG2a or CII-specific IgG2a were measured in serum obtained from both groups of mice by ELISA. (F) T cell proliferative responses were determined in a mixed lymphocyte reaction by a [3H]thymidine incorporation assay Data represent the mean ± SD of three independent experiments or representative of more than three independent experiments (*P<0.05, **P<0.01 and ***P<0.001).

We first investigated whether treatment with EGCG would suppress the arthritic inflammation and joint destruction in CIA mice of IL-1Ra deficient BALB/c background. Results showed a reduction of the arthritic score and arthritis incidence when the mice were treated with an intraperitoneal injection of EGCG (40 mg/kg) when compared to the vehicle injection (Figure 1A). Histological examination demonstrated that the arthritic joints of the EGCG treated mice had a lower degree of inflammation and cartilage damage compared to the vehicle treated mice (Figure 1B). The number of TRAP-positive cells, which were regarded as osteoclasts, was markedly lower in the arthritic joints of EGCG treated mice than those of vehicle treated mice (Figure 1C). To confirm the suppressive effect of EGCG on osteoclastogenesis *in vitro*, the isolated BMM cells were differentiated into osteoclasts with M-CSF and RANKL in the presence or absence of EGCG at various concentrations. As shown in Figure 1D, the addition of EGCG during the induction of osteoclastogenesis significantly inhibited osteoclast formation in a dose-dependent manner. Consistently, the serum levels of the IgG2a and CII-specific IgG2a was significantly lower in the mice treated with EGCG and T cell proliferation, represented by the [3H] thymidine incorporation assay, was markedly suppressed in T cells obtained from EGCG treated mice compared to those from vehicle treated controls (Figure 1E and F)

### EGCG attenuates the expression of inflammatory cytokines and molecules associated with mTOR signaling pathway in the arthritic joints

**Figure 2:**
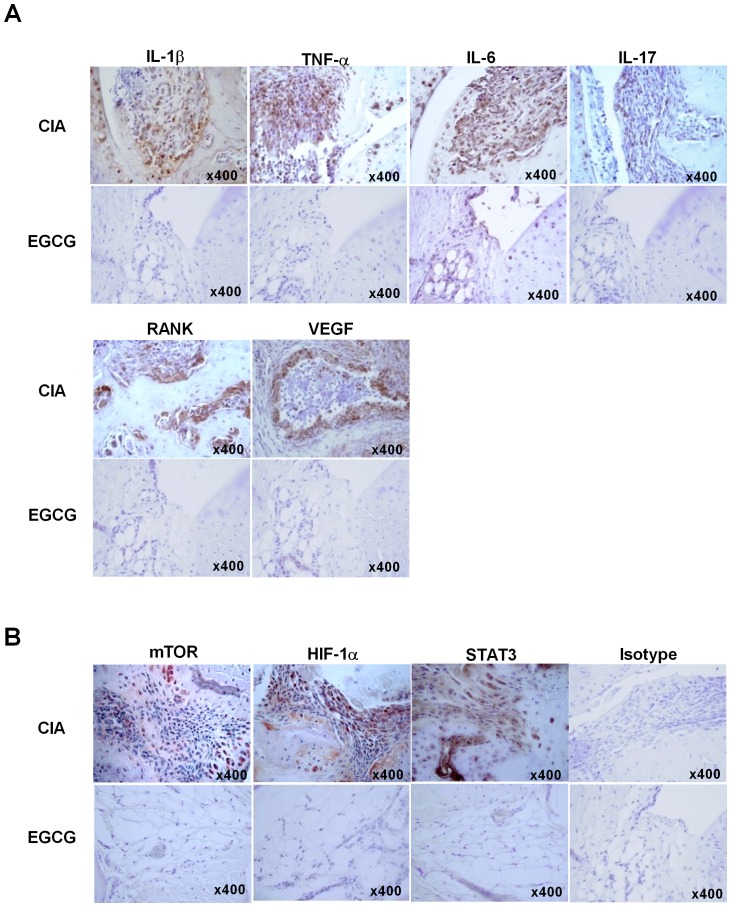
Expression of inflammatory cytokines and molecules associated with mTOR signaling. (A–B) The joint tissues were obtained from CIA and EGCG injected mice and were stained with the anti-IL-1β, anti–IL-6, anti-TNF-α, anti-IL-17, anti-RANK, anti-VEGF (A), anti-mTOR, anti-HIF-1α, anti-STAT3 or isotype control Abs (B). Data are representative of three independent experiments.

The anti-arthritic effect of EGCG was further supported by the results of IHC staining for proinflammatory cytokines that were implicated in RA pathogenesis. The expression of IL-1β, IL-6, TNF-α and IL-17 was markedly reduced in the joints of EGCG treated mice compared to those of vehicle treated mice. We also confirmed a reduction in RANK expression explaining the diminished number of osteoclasts. The expression of VEGF, the representative molecule of angiogenesis, was also suppressed with EGCG treatment (Figure 2A). Noteworthy, EGCG suppressed the expression of molecules associated with mTOR signaling pathway, which had an important role in Th17 differentiation. The expression of mTOR, HIF-1α and STAT3 was significantly lower in the joints of EGCG treated mice (Figure 2B).

### The anti arthritic effect of EGCG in IL-1RaKO CIA mice is dependent on the inhibition of IL-1β signaling and Th17

**Figure 3:**
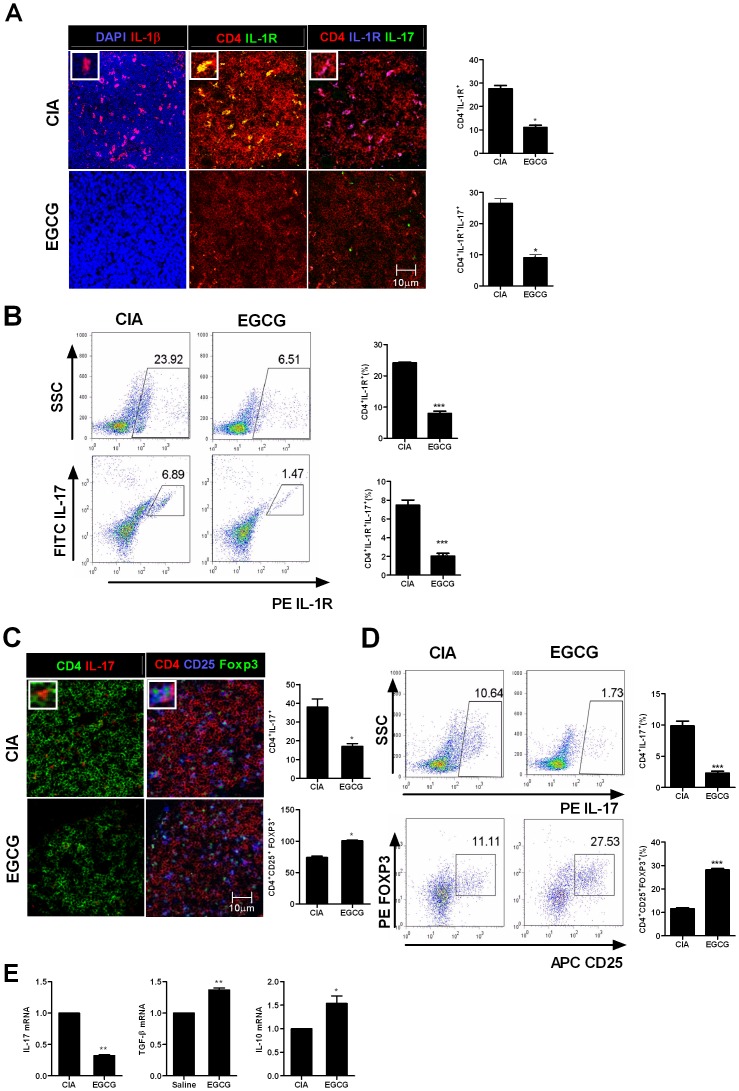
The anti arthritic effect of EGCG is dependent on the inhibition of IL-1β signaling and IL-17 expression. (A) Spleen tissues from each mouse were analyzed for CD4, IL-17 (green), IL-1R (green or blue), IL-1β (red) and DAPI by confocal microscopy.(B) Splenocytes were stained with CD4-PerCP, IL-17-FITC, and IL-1R-PE using flowcytometry. (C) CD4^+^CD25^+^Foxp3^+^ T cells and CD4^+^IL-17^+^ T cells using Abs specific for CD4 (red), CD25 (blue), Foxp3 (green) and IL-17 (red). (D) Splenocyteswere stained with CD4-PerCP, CD25-APC, Foxp3-PE and IL-17-FITC.(E) The expression of Foxp3, IL-17, IL-10 and TGF-β were determined by real-time PCR. Data represent the mean ± SD of three independent experiments or representative of more than three independent experiments (*P<0.05 and **P<0.01).

As augmented IL-1β signaling contributes to more severe arthritis in IL-1RaKO mice, we investigated whether the suppressive effect of EGCG was associated with inhibition of IL-1β signaling. As shown in Figure 3A and B immunostaining and FACs staining of the spleens from EGCG-treated mice showed a reduction in the number of IL-1β positive cells. Moreover, the number of CD4^+^IL-1R^+^ T cells also decreased with EGCG treatment, suggesting IL-1β signaling might be diminished in EGCG treated mice. As a result, the number of CD4^+^IL-1R^+^IL-17^+^ T cells also reduced (Figure 3A, B). Consistently, the numbers of CD4^+^IL-17^+^ T cells in the spleens were lower in EGCG treated mice than the controls. Alternatively, the numbers of CD4^+^CD25^+^Foxp3^+^ splenocytes were greater in the EGCG-treated group (Figure 3C,D). The regulatory effect on Th17/Treg was also demonstrated by the degree of mRNA expression of the associated molecules. The mRNA expression of IL-17 was reduced as IL-10 and TGF-β was enhanced in the splenocytes of EGCG treated mice (Figure 3E).

### EGCG regulates Th17 via inhibiting mTOR, HIF-1α, and STAT3

**Figure 4:**
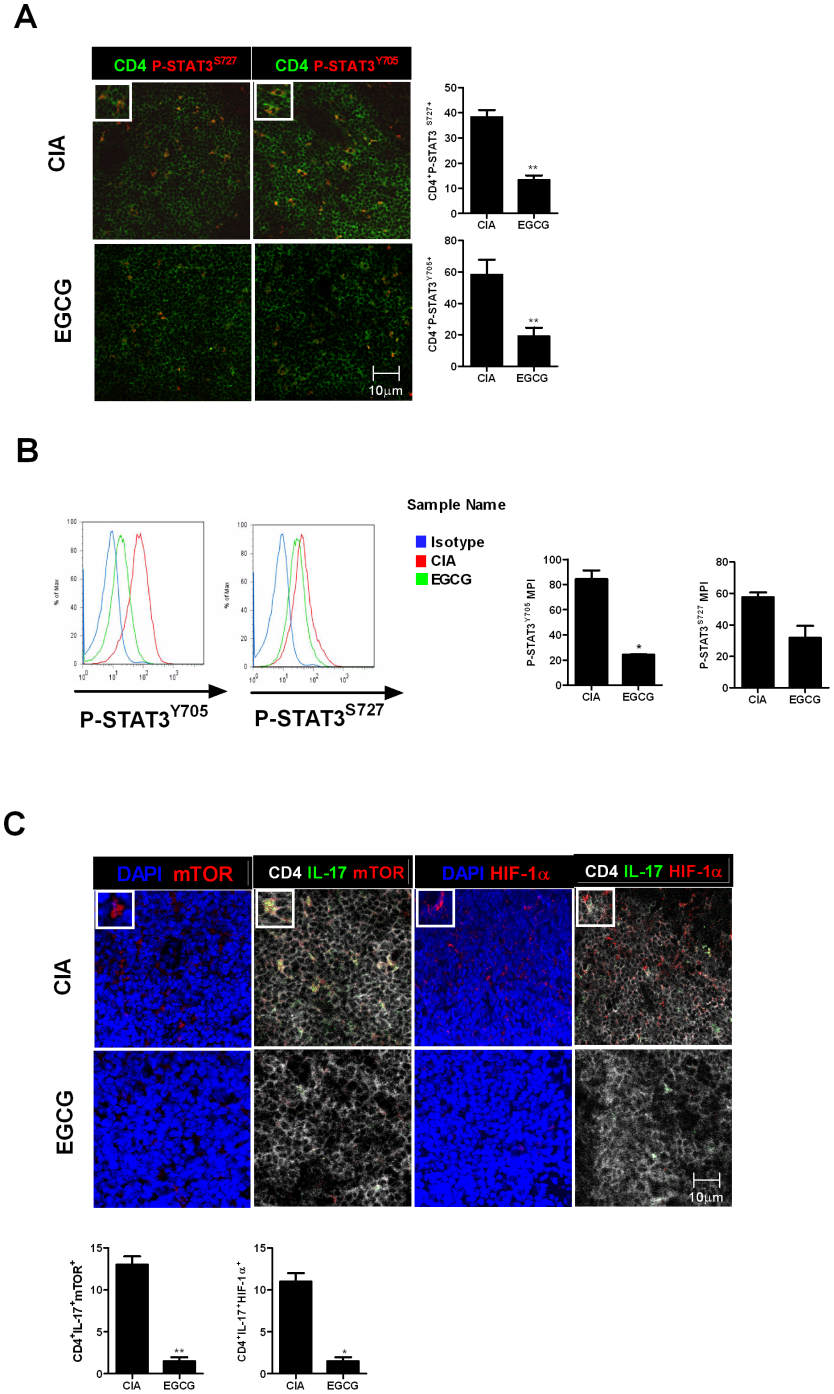
EGCG decreased signaling of associated Th17 differentiation. (A) Spleen tissues from each mouse were stained for *p*-STAT3 (S727) (red), *p*-STAT3 (Y705) (red) and CD4 (green).(B) For flowcytometry analysis, splenocytes were stained with CD4-PerCP,*p*-STAT3705-PE or *p*-STAT3727-PE. (C) Spleen tissues from each mouse were stained for CD4 (white), IL-17 (green), mTOR (red), HIF-1α (red) and DAPI. Data are representative of more than three independent experiments (*P<0.05 and **P<0.01).

**Figure 5:**
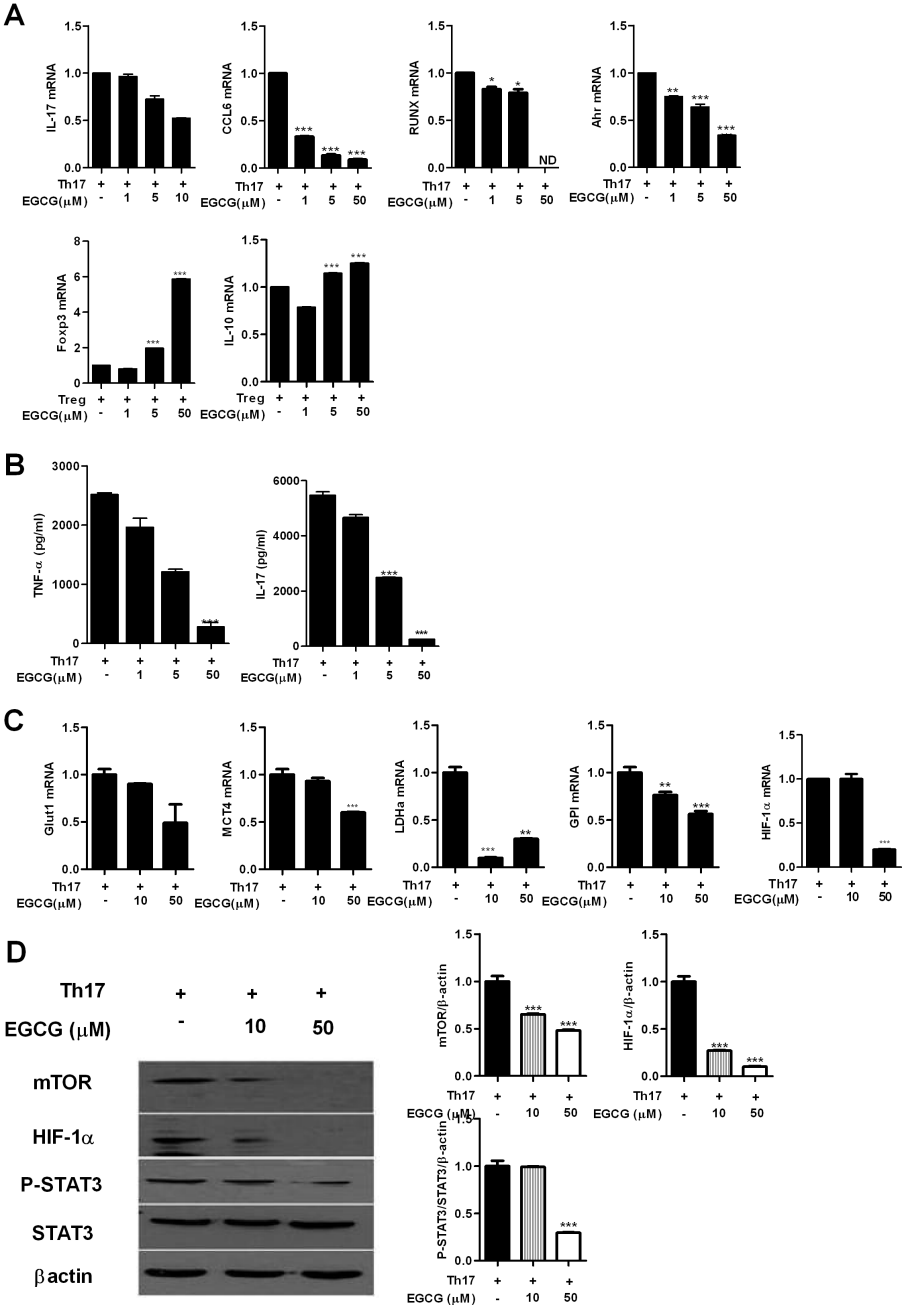
EGCG decreases the frequency of Th17 cells via inhibition mTOR, HIF-1α and STAT3. (A) The expression levels of IL-17, CCL6, RUNX, AHR, Foxp3 and IL-10 by real-time PCR in murine splenocytes. (B) IL-17 and TNF-α were measured in the culture supernatant by ELISA. (C) The expression levels of Glut-1, LDHα, MCT4, GPI and HIF-1α by real-time PCR. (D) Expression of *p*-STAT3, HIF-1α and mTOR in splenocytes was examined by western blot. Data represent the mean ± SD of three independent experiments or representative of more than three independent experiments (*P<0.05, **P<0.01 and ***P<0.001).

As marked reduction in IL-17 and mTOR signaling molecules was observed in the joints of EGCG treated mice, we investigated whether this could explain the altered numbers of Th17 and Treg cells using confocal microscopy and flowcytometry As shown in Figure 4A,B, EGCG treatment reduced the number of CD4+*p*-STAT3+ T cells in the spleens of the CIA mice. And the expression of mTOR and HIF-1α was decreased in IL-17 producing CD4 T cells with EGCG treatment (Figure 4C). These data collectively suggest that the inhibition of mTOR, HIF-1α and *p*-STAT3 contributed to a reduction of Th17 cells in the CIA mice. To confirm the inhibitory effect on Th17 differentiation, mice splenocytes were cultured in Th17 polarizing condition with various concentrations of EGCG. Real-time PCR revealed that the expression of molecules associated with Th17 including IL-17, chemokine (C-C motif) ligand 6 (CCL6), runt-related transcription factor (Runx) and aryl hydrocarbon receptor (Ahr) was diminished while the mRNA levels of Treg associated molecules such as Foxp3 and IL-10 increased in a dose-dependent manner (Figure 5A). The level of TNF-α and IL-17 was consistently lower in the culture supernatant of EGCG-treated cells (Figure 5B). Similar to the *in vivo* results of CIA mice, inhibition of *in vitro* Th17 differentiation was also dependent on mTOR, HIF-1α and *p*-STAT3 (Fig 5D). The markers of glycolysis, HIF-1α, GLUT-1, MCT4, LDH-α and GPI which favor Th17 differentiation were suppressed with EGCG treatment (Figure 5C).

## Discussion

In the present study, we demonstrated that treatment with EGCG suppressed the CIA in IL-1Ra deficient mice. EGCG decreased the expression of IL-1R in CD4+ T cells and suppressed HIF-1α, which supposedly drives the metabolism toward a Th17 favoring condition resulting in reduced Th17 and enhanced Treg. In addition, EGCG negatively regulated osteoclastogenesis both *in vivo* and *in vitro*. These seem to collectively contribute to the suppression of CIA.

IL-1RaKO mice that used in our experiments lack the IL-1β receptor antagonist, which blocks IL-1β, and develop spontaneous arthritis as they age [7]. To enhance the severity of arthritis, CIA was induced in this strain. Consistent with previous CIA induced in DBA/1 mice [5], our results showed that EGCG treatment diminished the expression of inflammatory cytokines and the serum level of CII-specific antibody. This study also demonstrated the inhibitory effect of EGCG on osteoclastogenesis both *in vivo* and *in vitro*. Lin *et al.* suggested that EGCG inhibited osteoclastogenesis by hampering RANKL-induced NFκB activation while Morinobu *et al.* reported that EGCG down-regulated the expression of NFATc1, but not of NFκB, c-Fos and c-Jun [8]. Despite these inconsistent mechanisms, it is clear that EGCG inhibited osteoclastogenesis. In arthritic joints, osteoclastogenesis is largely dependent on RANKL expression and was recently reported that IL-1β and TNF-α initiated IL-6-STAT3 pathway was critical in RANKL expression in inflammatory arthritis [9]. Our data showed EGCG suppressed IL-1β, TNF-α, IL-6 and STAT3, which seemed to contribute to the decreased osteoclastogenesis as well. Both inhibitory mechanisms of up- and down-stream of RANK-RANKL signaling pathway seem to have rendered the regulation of EGCG.

As the pathogenesis of RA emphasizes the critical role of Th17, the effect of EGCG on Th17 was studied. Results showed that Th17 differentiation was diminished with EGCG treatment while enhancing Treg differentiation. One explanation for this is the effect of reduced IL-1R expression. We demonstrated that EGCG down-regulated IL-1R expression in CD4+ T cells, which was consistent with previous reports that EGCG reduced IL-1R expression in the pancreatic adenocarcinoma cells [10], although a different cell type. However, the proinflammatory effect of IL-1β, IL-1β signaling through T cell-specific IL-1R is known to promote Th17 differentiation [11]. EGCG treated mice showed reduced expression of IL-1β both in the arthritic joints and splenocytes. Decreased level of IL-1β and T cell specific IL-1R seem to synergistically act to reduce Th17 differentiation.

Wu *et al* demonstrated that EGCG negatively regulated Th1 and Th17 while enhanced Treg [12]. They suggested that EGCG suppressed the expression of the essential transcription factors; T-bet (Th1), STAT3 (Th17) and RORγt (Th17) for T cell differentiation. In regards to Treg, they advocated that natural Treg was unaffected while induced Treg slightly increased. The reciprocal regulation of Th17 and Treg may result from the plasticity of two cell types. The decreased level of *p*-STAT3 by EGCG treatment seems to lead Treg polarizing condition. Also, another mechanism has been proposed showing that EGCG enhanced the soluble gp130 receptor for IL-6 and suppressed IL-6 signaling for Th17 differentiation [13].

This study focused on the role of HIF-1α in Th17 regulation where results demonstrated HIF-1α is induced in hypoxic condition and enhances angiogenesis. It was recently suggested that this is a metabolic check point for the differentiation of Th17 and Treg cells [14]. Glycolysis is dominant in Th17 cells and the expression of molecules associated with glycolysis including GLUT-1, MCT4, LDH-α and GPI. On the other hand, Treg cells use lipids as their energy source. Thus, the expression of glycolysis-associated molecules is essential for Th17 differentiation and the expression of these molecules is dependent on HIF-1α, making it the ‘check point’. In addition, HIF-1α directly upregulates RORγt transcription and forms a complex with p300 and RORγt to bind to the IL-17 promoter to enhance its transcription. Conversely, it binds to Foxp3 and leads it proteasomal degradation, resulting in Treg suppression [15]. It was previously reported that EGCG suppressed HIF-1α expression in human skin and nasal polyp fibroblasts [16], [17]. Consistent with previous reports, our data demonstrated that the expression of glycolysis-associated molecules as well as HIF-1α increased when Th17 was enhanced, which was abolished by EGCG treatment. We also demonstrated that the inhibitory effect of EGCG on HIF-1α was mediated by the upstream suppression of mTOR, which was required in HIF-1α dependent Th17 differentiation [3]. Other studies have illustrated that EGCG could inhibit mTOR activation by suppressing PI3-Akt pathway or activating AMPK [18]–[20]. Although the upstream of mTOR in our data was not demonstrated, it is clear that down-regulation of mTOR-HIF-1α by EGCG contributed to reduction in Th17 and consequent amelioration of arthritis.

EGCG demonstrated therapeutic effects in Th17-associated diseases such as experimental autoimmune encephalitis [21] and inflammatory bowel diseases [22], [23] as well. It seems that the suppressive effect on Th17 also worked in those diseases. EGCG has also shown to inhibit vascular inflammation and atherosclerosis [24]. Given that cardiovascular comorbidity is of great concern in treating RA, EGCG seems to be a promising therapeutic agent.

In conclusion, EGCG suppressed CIA in IL-1Ra deficient mice. The diminished expression of IL-1R in CD4+ T cells with EGCG treatment seems to have contributed to the reduction of Th17 differentiation. More importantly, EGCG suppressed the activation of mTOR and subsequently HIF-1α, which is considered as a metabolic check point of Th17/Treg differentiation. The authors suggest a novel metabolism-associated regulation of Th17 by EGCG in CIA model, providing the support of a therapeutic potential of EGCG in autoimmune arthritis.
